# The insomnia phenotype in genetic Creutzfeldt–Jakob disease based on the E200K mutation

**DOI:** 10.1080/19336896.2019.1590938

**Published:** 2019-03-28

**Authors:** Eva Feketeova, Dominika Jarcuskova, Alzbeta Janakova, Marianna Vitkova, Jozef Dragasek, Zuzana Gdovinova

**Affiliations:** a Department of Neurology, P.J. Safarik University, Kosice, Slovakia; b Department of Neurology, University Hospital of L. Pasteur, Kosice, Slovakia; c 1st Department of Psychiatry, University Hospital of L. Pasteur, Kosice, Slovakia; d Department of Prion Diseases, Slovak Medical University, Bratislava, Slovakia; e 1st Department of Psychiatry, University of P. J. Safarik, Kosice, Slovakia

**Keywords:** Insomnia, genetic Creutzfeldt–Jakob disease, E200K, polysomnography, prion diseases

## Abstract

The aim of the presented study was to reveal the frequency of insomnia spells in E200K genetic Creutzfeldt–Jakob disease (gCJD) patients. Clinical records of 22 subjects diagnosed with E200K gCJD were retrospectively reviewed. The patients w/wo insomnia (n = 4, 18%/n = 18, 82%) did not differ in age, sex and the duration of the symptomatic phase. Analysis of the clinical features in the groups yielded differences in the clinical signs in the early phase of the disorder, proportion of homozygotes (Met/Met) at codon 129, MRI changes in the thalamus and the typical EEG abnormality. The study suggests that apart from traditional clinical features, the insomnia is not a rare early symptom in phenotype of E200K gCJD based on early thalamic involvement.

Genetic prion diseases are fatal encephalopathies caused by mutations in the prion protein gene (PRNP). Genetic forms of prion diseases account for about 10–15% of cases [1–3]. Over 60 pathogenic PRNP mutations have been published. Their frequency varies in different populations, and just five of them are responsible for about 85% of genetic prion diseases around the world: E200K, V210I, V180I, P102L and D178N [4]. Traditionally, genetic prion diseases were classified into three forms: familial Creutzfeldt–Jakob disease (fCJD), Gerstmann–Sträussler–Scheinker disease (GSS) and Familial Fatal Insomnia (FFI). All of them were characterized as human fatal spongiform encephalopathies, presenting with dementia, myoclonus, ataxia, extrapyramidal/pyramidal signs and symptoms and akinetic mutism in the case of CJD; cerebellar ataxia, gait abnormalities, dementia, dysarthria, ocular dysmetria, infrequent myoclonus, spastic paraparesis, parkinsonian signs in the case of GSS; insomnia, dysautonomia and motor deficits in the case of FFI. They are also typically accompanied by specific changes in EEG and brain MRI. Apart from these typical features, some patients present with unusual symptoms, such as a stroke-like presentation, alien hand syndrome, visual disturbances [5], spastic paraparesis [6] and others. Specific phenotypes and unusual symptoms could overlap in different PRNP mutations, or the same PRNP mutation could differ in phenotype: the D178N mutation could present as gCJD or FFI, determined by the type of codon 129 polymorphism; or the D178N–129M haplotype, expressing the FFI phenotype, corresponds to MM2-Thalamic (MM 2T or sporadic FI) in sporadic CJD (sCJD) [7].

Slovakia is a unique region in Europe with a large cluster of gCJD based on the E200K mutation (70–80% of all CJD cases are gCJD) [8]; FFI has never been diagnosed, and features of its phenotype have been reported among gCJD cases. The purpose of our study was to explore the frequency of insomnia spells in gCJD based on E200K mutation and to review whether gCJD cases with insomnia could represent a specific phenotype variant with specific clinical electrophysiological, and neuroimaging features.

## Methods

Clinical records of 22 subjects diagnosed with gCJD (based on E200K) between 1 January 2004 and 31 July 2017 were reviewed. Demographic and general clinical data, laboratory findings, results of electrophysiological examinations and MRI scans were analysed retrospectively. Age at onset, disease duration in months, codon 129 polymorphism (Met/Met, Met/Val) and protein 14–3–3 in the CSF were extracted from the medical records. The subjects were divided into the groups w/wo insomnia based on detailed analysis of the subjective spells, objective neurological examinations, with a special focus on onset symptoms of the disorder.

Descriptive statistics were calculated for every group. Significances (P) were tested by statistical software program PASW SPSS version 21.0 for Windows (SPSS Inc, Chicago, IL).

Ethics committee approval was not needed based on local legal standards.

## Results

For statistical analysis, the study population was divided into two groups based on the presence of insomnia among the initial spells: Group I – patients complaining of insomnia during their first visit: n = 4 (18%), mean age 63 ± 7yy, and Group II – patients without a complaint of insomnia: n = 18 (82%), mean age 58 ± 7yy.

Group I patients were older, though the difference was not statistically significant, and the male/female proportion did not vary. All the patients in Group I were homozygots (Met/Met) on codon 129 in comparison with 61% of Group II patients. Protein 14–3–3 in CSF was present in 60–71% in both groups; details in Table 1.

**Table 1.** Demographic data with the results of genetic testing and the presence of protein 14–3–3 in CSF for gCJD cases with insomnia (Group I) and without insomnia (Group II).

		Group I	Group II	Statistical significance
n		4	18	
Age of Onset	Mean ± SD YY	63 ± 7	58 ± 7	P = NS
	Median YY	66	56	
Sex	Male/Female	1/3	8/10	P = NS
Duration of the Symptomatic phase MM (median)		6.3 ± 3.8 (5.5)	5.6 ± 4.7 (4)	P = NS
Genetic testing				
E200K (%)		4 (100)	18 (100)	
Met/Met (%)		4 (100)	11 (61)	P = NS
Met/Val (%)		0 (0)	7 (39)	
Val/Val (%)		0 (0)	0 (0)	
CSF: Protein 14–3–3				
Present (%)		2 (50)	14 (78)	P = NS
Not present (%)		2 (50)	4 (22)	
Not available (%)		0 (0)	1 (6)	

Eleven gCJD patients (50%) came from eight families having at least two members with gCJD (E200K). Three out of four cases in Group I (75%) had the familial form of the disorder. The proportion of familial gCJD cases in Group II was 44% (eight patients).

**Figure 1.**
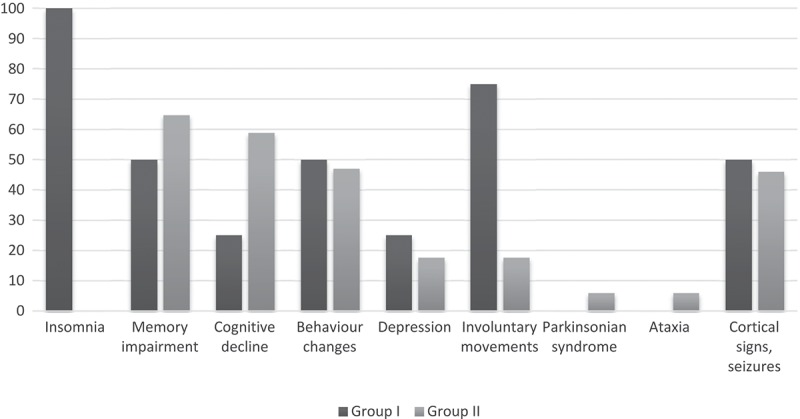
Frequency of early symptoms of gCJD cases with insomnia (Group I) and without insomnia (Group II).

The duration of the symptomatic phase of gCJD was longer in insomnia patients (median 6.3/5.6 months), though the difference was not significant. Analysis of the clinical features yielded less frequent mental impairment (memory, cognitive decline, disorientation) in the early phase in Group I, as well as increased mood disturbance, emotional lability and prominent involuntary movements and motor disability in Group I (Figure 1).

**Figure 2.**
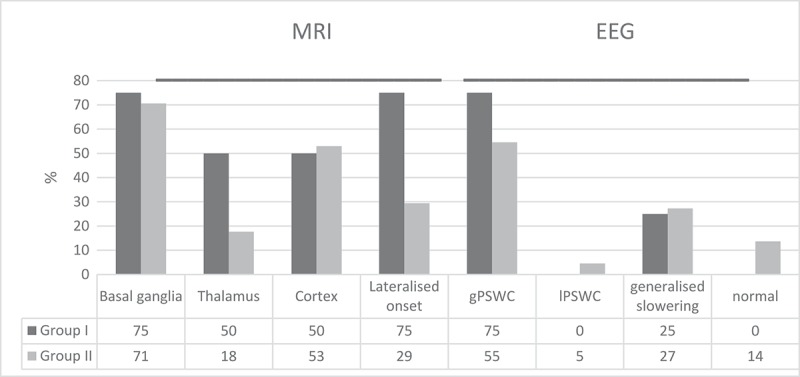
Localization of MRI (T2 and DWI sequences) and type of EEG changes in gCJD cases with insomnia (Group I) and without insomnia (Group II). (gPSWC – Generalized Periodic Sharp Wave Complexes, lPSWC – Lateralized Periodic Sharp Wave Complexes).

A brain MRI was available for visual retrospective analysis in 16 of 22 patients: all from Group I and 12 from group II (Figure 2). Pathological changes on the T2, FLAIR and DWI sequences of the MRI were increased in the thalamus, and the changes were frequently lateralized in Group I. Electroencephalographic findings of typical generalized periodic sharp wave complexes (gPSWC) were more frequent in Group I.

**Figure 3.**
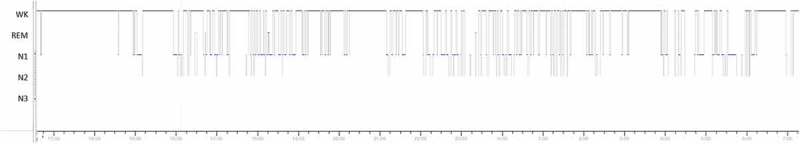
Hypnogram of a 51-year-old male familial gCJD E200K patient with prominent insomnia; monitoring was set approx. 8 months after the onset of insomnia and depressive symptoms. It shows fragmented night sleep shifting between NREM 1 (N1), NREM 2 (N2) and Wakefulness (WK) and the intrusion of sleep into the wakefulness.

One patient with insomnia underwent polysomnographic recording. Monitoring for 18.7 h showed fragmented sleep with low efficiency, absence of deep sleep stages (NREM 3 and REM) and a very low frequency of sleep spindles and K-complexes during NREM 2. Total sleep time was 6 h, and circadian disruption was shown with the intrusion of light sleep into wakefulness and wakefulness into sleep. No sleep-related breathing disorder was present; the index of periodic leg movements was within the normal range, and tremor during wakefulness registered on the lower limbs showed a frequency of 3–4 Hz. Figure 3 shows the hypnogram.

## Discussion

The presented study of 22 E200K gCJD patients yielded insomnia accompanied by depressive symptoms and/or emotional lability and/or unspecific headache as early symptoms of CJD in 18% of patients. Genetic testing revealed methionine homozygosity at codon 129 in all of them. gCJD patients with insomnia had less prominent motor symptoms and memory/cognitive deterioration in the early phase, and the involvement of the thalamus on MRI was much more frequent, as was the presence of typical gPSWC in EEG.

Insomnia and Clinical features

The first symptoms of CJD are non-specific: fatigue, dizziness, forgetfulness, irritability and personality changes. In the absence of more specific CJD symptoms, such as rapid progressive dementia with ataxia, myoclonus and pyramidal and extrapyramidal motor signs, the diagnosis of CJD could be difficult. A rare phenotype, known as fatal insomnia and including sleep disturbance, distal pain/sensory disturbance and abnormalities of the autonomic nervous system, is typically associated with the D178N missense mutation of PRNP and methionine homozygosity at codon 129.

The frequency of insomnia in autopsy-verified CJD cases in the study by Kang et al. (24.6%) was similar to our cohort (18%), despite the fact that Kang evaluated different types of CJD, while our patients were gCJD E200K. What’s more, our results did not vary, even when we evaluated retrospective data in the records, while Kang obtained sleep history based on a somnologist prospective evaluation [9].

Similarly, Wall et al. [10] identified sleep symptoms in 62 of 126 patients (49%) in definite or probable CJD, insomnia itself in 31 (25%), and the majority of the sleep disturbances (79%) occurred within the first 100 days following disease onset. It was not possible to identify the source of the data in the study (retrospective analysis of patients records, somnologist examination). Surprisingly, there was no significant difference in insomnia frequency across the studies, even when the data were obtained in prospective or retrospective study analysing only data in the clinical records or carriers. A cognitive impairment, present in the early stages of CJD, probably influenced the clinical history data taken from the patients themselves, and the sensitivity of such analysis is comparable with a retrospective one, as was used in our study.

The clinical symptoms of all presented cases belonged to the classical CJD phenotype; however, separate analysis of the cases with insomnia in the early phase brought less frequent mental impairment, increased mood disturbance, emotional lability and prominent involuntary movements (tremor, dystonia) in a comparison with cases without insomnia, which showed more pronounced mental impairment, motor disability and epileptic seizures since the onset of the disorder. The phenotype of insomnia CJD E200K patients in our study was clearly distinguishable from the classical form without insomnia in the early phase, as has been published in just single cases up to date [11,12]. The retrospective nature of our study did not allow for detailed analysis of autonomic features as a component of the FFI phenotype.

Familial gCJD, Sex

PRNP gene penetration, expressed as the familial form of gCJD in the group without insomnia (50%), was in accordance with data previously published by Kovacs [2]. Familial cases in the group with insomnia was higher and covered 75%. Women were more susceptible in both groups, similar to other PRNP mutations [2].

Phenotypic variability E200K PRPN mutation, and codon 129 polymorphism

Clinical symptoms in insomnia E200K gCJD patients in the presented study strongly resemble thalamo-olivary (MM 2T) subtype of sCJD. Insomnia in that subtype of sCJD similar to our study is accompanied by psychomotor hyperactivity and ataxia [13]. Both groups – sCJD subtype 13] and insomnia E200K gCJD cases in the presented study – reveal methionine homozygosity. The pathological and clinical features of all forms of human prion disease are severely influenced by the codon 129 polymorphism. Our findings, showing that insomnia gCJD cases form just 27% of E200K methionine homozygotes and 18% of any E200K cases, point at the role of additional polymorphisms in phenotype of E200K gCJD. Insomnia phenotype in E200K gCJD is not rare compared to sCJD thalamo-olivary subtype presented in approx. 1% of sCJD [13]. Fatal insomnia based on D178N mutation presents just in methionine homozygotes, while heterozygotes of the mutation carriers show different phenotypes. It can be hypothesized that E200K PRPN mutation itself plays the role in the insomnia phenotypic variability, too. Unfortunately, the database set in 2004 does not allow us to run detailed additional genetic testing of the old cases in our study.

Protein 14–3–3

Clinical suspicion of prion disorder could be supported by testing CSF for protein 14–3–3 electrophysiological and radiological findings. The presence of 14–3–3 protein was more sensitive for the diagnosis of gCJD in the patients without insomnia in our sample. The detection of tau, phosphorylated tau protein, amyloid beta 1–42, S100B and neuron-speciﬁc enolase in CSF has not been done in the presented study and might explain phenotype differences in gCJD E200K cases in the future similar to molecular subtypes in sCJD [14].

MRI

Changes in brain MRI are an important diagnostic modality in rapid progressive dementias, even if their sensitivity and specificity vary across CJD subtype and the course of the disorder. The latest added sequences for CJD MRI protocol, such as FLAIR and DWI, increase MRI sensitivity to 90%, and DWI benefits from greater inter-observer agreement compared with FLAIR or T2 [15]. The increased signal in the MRI-DWI caused by a restriction of the diffusion of water protons within tissue is attributed to spongiform degeneration in sporadic CJD [16], while changes in the FLAIR sequences result from astrocytosis [17]. Meissner et al. [18], using MRI, detected a cortical signal increase and hyperintensities in the basal ganglia and thalamus across all molecular sporadic CJD subtypes. The findings suggest that characteristic MRI lesions may occur for each molecular subtype. Serial DWI in the cases of Oppenheim showed a progressive disappearance of the DWI hypersignal of the basal ganglia, replaced by pronounced atrophy believed to be related to progressive neuronal death in iatrogenic CJD [19].

Our findings, with the presence of FLAIR and DWI hyperintensities in basal ganglia of over 70% in both groups of gCJD E200K, slightly exceed the 58% proportion in the study of 20 German gCJD E200K. Other temporal abnormalities were not analysed in that study [20].

Detailed computerized volumetric analysis of brain MRIs revealed monotonic volume reductions in the thalamus and basal ganglia and lateral ventricles dilatation after clinical conversion in carriers of the E200K mutation [21]. Even more, thalamo-striatal diffusion reductions precede disease onset in PRNP mutation carriers [22]. A common role of the thalamus in CJD and FI was suggested based on volumetric and DTI changes [23]. Thalamic changes were not attributed to the specific phenotype differences in the above-mentioned studies. Our findings of more frequent thalamic hyperintensities in insomnia cases could point to the specificity of the insomnia phenotype of CJD E200K. On the other hand, we cannot exclude the role of technical issues of MRI (different sensitivities of the 1.5T and 3.0T equipment across the cases, and adding DWI sequences to the MRI protocol just in recent years), which could influence the proportion of thalamic changes in the insomnia group (all four patients were diagnosed in 2015 and 2016).

EEG, PSG

Several reports have suggested that PSWC correlate with thalamic involvement in CJD [24,25]. PSWC were present in 75% of our insomnia cohort versus 55% in the group without insomnia. The frequency of PSWCs in different populations of gCJD E200K varies from 43% to 65% to 74% [2,26,27], describing both our groups within this range, despite the fact that the intergroup comparison shows higher frequency in insomniacs.

Contrary to the EEG and MRI findings in the previous studies in thalamo-olivary sCJD [13], most of the insomnia gCJD cases showed gPSWCs, half of them elevated protein 14–3–3 and thalamic involvement without sparing basal ganglia in MRI picture, suggesting unique insomnia phenotype in gCJD E200K.

A single polysomnographic finding in a patient with insomnia is consistent with the sleep evaluation of the patients with sporadic and familial FI [28,29]; however, sleep disordered breathing was not present in our case, nor were abnormal movements. The relatively longer total sleep time in our case is probably due to the longer monitored time showing sleep fragmentation and light sleep intrusion into the wakefulness.

In summary, we have provided the ﬁrst detailed clinical analysis of gCJD E200K patients with the insomnia phenotype, accompanied by milder cognitive impairment and increased frequency of abnormal movements, less expressed motor and gait features in early phase in comparison with the classical gCJD phenotype. MRI findings showing frequent thalamic hyperintensities, EEG with more frequent PSWC abnormalities in insomnia group support early thalamic involvement in one-fifth of gCJD E200K patients expressed by alternative insomnia phenotype. The size of the sample and retrospective nature of the study did not allow risk factors for the insomnia phenotype in gCJD E200K to be detected, apart from methionine homozygosity on 129 codon, which is a task for further evaluation.
